# Synthesis and Preclinical Evaluation of Radio-Iodinated GRPR/PSMA Bispecific Heterodimers for the Theranostics Application in Prostate Cancer

**DOI:** 10.3390/pharmaceutics11070358

**Published:** 2019-07-23

**Authors:** Ayman Abouzayed, Cheng-Bin Yim, Bogdan Mitran, Sara S. Rinne, Vladimir Tolmachev, Mats Larhed, Ulrika Rosenström, Anna Orlova

**Affiliations:** 1 Department of Medicinal Chemistry, Uppsala University, 75183 Uppsala, Sweden; 2 Department of Immunology, Genetics and Pathology, Uppsala University, 75183 Uppsala, Sweden; 3 Science for Life Laboratory, Uppsala University, 75183 Uppsala, Sweden

**Keywords:** prostate cancer, GRPR, PSMA, bispecific heterodimers, theranostics, radio-iodine

## Abstract

Gastrin-releasing peptide receptor (GRPR) and prostate-specific membrane antigen (PSMA) are overexpressed in most prostate cancers. GRPR expression is higher in early stages while PSMA expression increases with progression. The possibility of targeting both markers with a single theranostics radiotracer could improve patient management. Three GRPR/PSMA-targeting bispecific heterodimers (urea derivative PSMA-617 and bombesin-based antagonist RM26 linked via X-triazolyl-Tyr-PEG2, X = PEG2 (BO530), (CH_2_)_8_ (BO535), none (BO536)) were synthesized by solid-phase peptide synthesis. Peptides were radio-iodinated and evaluated in vitro for binding specificity, cellular retention, and affinity. In vivo specificity for all heterodimers was studied in PC-3 (GRPR-positive) and LNCaP (PSMA-positive) xenografts. [^125^I]I-BO530 was evaluated in PC-3pip (GRPR/PSMA-positive) xenografts. Micro single-photon emission computed tomography/computed tomography (microSPECT/CT) scans were acquired. The heterodimers were radiolabeled with high radiochemical yields, bound specifically to both targets, and demonstrated high degree of activity retention in PC-3pip cells. Only [^125^I]I-BO530 demonstrated in vivo specificity to both targets. A biodistribution study of [^125^I]I-BO530 in PC-3pip xenografted mice showed high tumor activity uptake (30%–35%ID/g at 3 h post injection (pi)). Activity uptake in tumors was stable and exceeded all other organs 24 h pi. Activity uptake decreased only two-fold 72 h pi. The GRPR/PSMA-targeting heterodimer [^125^I]I-BO530 is a promising agent for theranostics application in prostate cancer.

## 1. Introduction

Prostate cancer remains one of the most commonly diagnosed and deadliest cancers in men despite the major advancements made to improve the survival and management of patients [1]. Despite significant progress, the conventional imaging approaches (based on ultrasound or magnetic resonance imaging (MRI)) do not allow for accurate staging of prostate cancer or the detection of its metastases with high sensitivity [2]. Although the advancements in management of patients with metastatic castration resistant prostate cancer have significantly improved the survival of patients, the improvement is limited to a few months [3]. Therefore, new approaches need to be investigated to further improve the detection and management of prostate cancer patients [4,5].

Several prostate cancer-associated molecular targets have been identified over the past few decades [6]. Two of the most investigated ones are the gastrin-releasing peptide receptor (GRPR), a G-protein-coupled receptor, and prostate-specific membrane antigen (PSMA), a type II transmembrane glycoprotein [6]. GRPR is naturally expressed in a number of organs including the pancreas and is overexpressed in several cancers such as prostate and breast cancers [7]. Several studies reported that 63%–100% of prostate cancer samples are GRPR positive [8,9,10,11]. The overexpression of GRPR is higher in the early stages of prostate cancers and decreases as the disease progresses [12]. PSMA is endogenously expressed in the kidneys and the prostate gland [13], and also overexpressed in several cancers including prostate cancer (three-order of magnitude higher than in normal prostate tissue). Its overexpression in prostate cancers is found in more than 95% of prostate cancer samples [14,15] and the expression increases as the disease progresses [16]. Moreover, PSMA overexpression was also found in the neovasculature of tumors and not in the normal vasculature [17,18].

Several radiotracers have been developed to target either GRPR or PSMA with the aim to detect and treat prostate cancer. Bombesin-based agonists and antagonists targeting GRPR, and derivatives of l-α-glutamyl-urea targeting PSMA have been studied in clinics [19,20]. RM2 and PSMA-11 were compared in the same patients for their sensitivities towards prostate cancer and its distant metastases [21]. Both tracers showed high sensitivity towards either GRPR or PSMA overexpressing cells, however lesions that could be detected just by one of the tracers were identified. This could be attributed to the different levels of GRPR or PSMA expression in some of the targeted regions.

These findings have made the concept of targeting both GRPR and PSMA with a single radiotracer attractive as it can cover the whole spectrum of prostate cancer and improve the imaging sensitivity towards prostate cancers. Moreover, synthesizing a bispecific heterodimer which can be used in theranostic applications will provide a more convenient approach to diagnose and manage patients with prostate cancers. Recently a few studies have been published describing the development and evaluation of bispecific tracers labeled with radiometals to target both GRPR and PSMA [22,23,24]. All reported heterodimers included the l-α-glutamyl-urea moiety to target PSMA and bombesin-based GRPR agonists.

**Figure 1:**
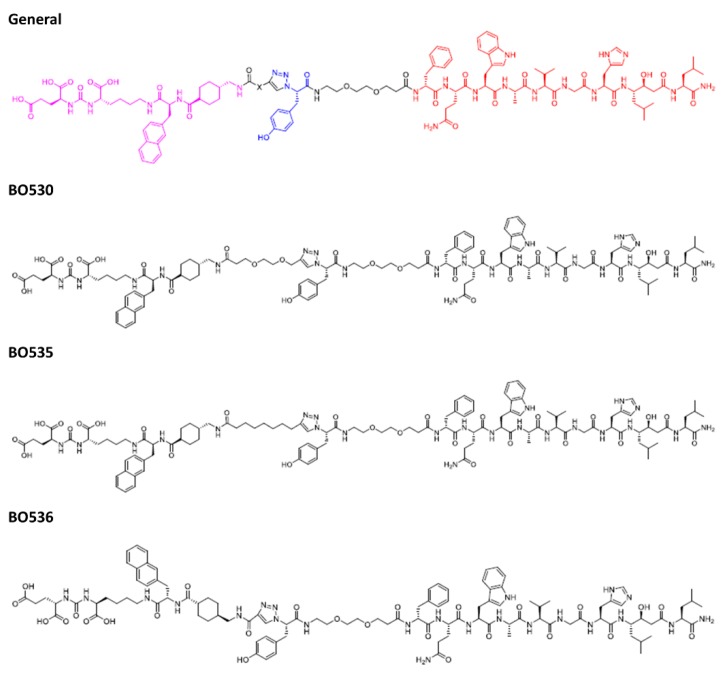
General structure of the heterodimers with a X indicating different linkers. The gastrin-releasing peptide receptor (GRPR) binding part is red, the prostrate-specific membrane antigen (PSMA)-binding part is magenta and the tyrosine for radio-iodonation is blue.

Previously, bombesin-based agonists to GRPR have been tested for imaging. Despite high tumor specificity, activation of GRPR triggers physiological responses and promotes tumor growth [15]. It was also demonstrated that agonists rapidly down regulate GRPR (10-fold within 10 min [25]) resulting in lower in vivo tumor activity uptake for agonists than for the antagonists [26]. A bombesin-based GRPR antagonist PEG*_n_*-RM26 has been extensively evaluated in our group and it was found suitable both for imaging and therapeutic application for GRPR-expressing prostate cancer. It demonstrated high affinity and specificity towards GRPR providing an imaging of xenografts with high contrast, and it inhibited tumor growth when administered with lutetium-177 [27,28,29]. PSMA-617, a l-α-glutamyl-urea derivative, has also been thoroughly investigated in preclinical studies demonstrating favorable kinetics [30] and is under evaluation in a number of clinical studies showing promising results [31,32]. We hypothesized that a hybrid of these two targeting molecules in a bispecific heterodimer could be used for a theranostic application in the management of prostate cancer. Herein we designed a set of heterodimers suitable for radio-iodination via tyrosine. Radio-iodination is suitable for theranostics agents due to the availability of different iodine isotopes: I-123 for SPECT, I-124 for PET, and I-131 for therapy. PSMA and GRPR targeting moieties were coupled via linkers with different lipophilicity via X-triazolyl-Tyr-PEG2 (X = PEG2 (BO530), (CH_2_)_8_ (BO535), or none (BO536)). The reported heterodimers possess GRPR antagonistic function by incorporating the GRPR antagonist, RM26 (d-Phe-Gln-Trp-Ala-Val-Gly-His-Sta-Leu-NH_2_) linked to the PSMA binding ligand PSMA-617 (Figure 1). The heterodimers were radio-iodinated on the tyrosine using a mild oxidant. The aim of this study was to evaluate in vitro and in vivo biological properties of the new radio-iodinated heterodimers.

## 2. Materials and Methods 

Prostate carcinoma cell lines PC-3 (GRPR positive), LNCaP (PSMA positive), and prostate carcinoma PC-3 cells transfected with PSMA PC-3pip (GRPR and PSMA positive) were maintained as described in Appendix A. All animal studies were approved by the Ethics Committee for Animal Research in Uppsala, Sweden following the national legislation on protection of laboratory animals (5/16, 26 February 2016). The activity content was measured using a 2840 automated gamma counter Wizard2TM (PerkinElmer, Norwalk, CT, USA). Statistical analyses were done by unpaired, two-tailed *t*-tests using GraphPad Prism 8 for Windows (GraphPad Software Inc, San Diego, CA, USA), *p* values below 0.05 were considered significant.

### 2.1. Syntheses of Heterodimers

**Scheme 1:**
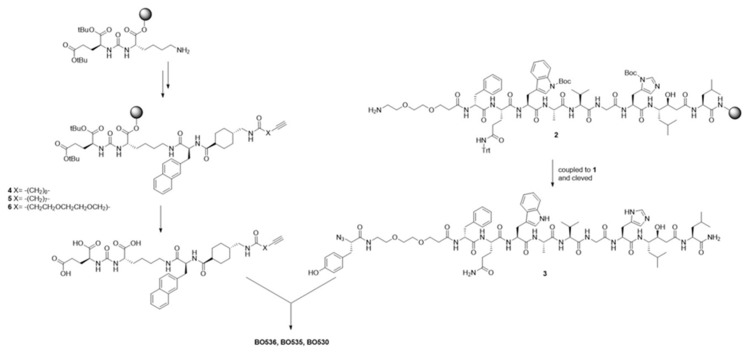
Synthesis scheme of heterodimers BO530, BO535, and BO536.

Chemicals and equipment used for syntheses are described in detail in Appendix A. Heterodimers BO530, BO535, and BO536 were synthesized according to Scheme 1.

#### 2.1.1. 2-Azido-l-Tyr(tBu)-OH (**1**)

Synthesis of 2-azido-l-Tyr(*t*Bu)-OH ((S)-2-azido-3-(4-*tert*-butoxyphenyl)propanoate) was performed according to previously described protocols [33]. All glassware was dried, and MeOH was dried over molecular sieves (3 Å). H_2_N-Tyr(*t*Bu)-OH (1 g, 4.1 mmol) was suspended in MeOH (100 mL). K_2_CO_3_ (2.75 equiv., 1.6 g, 11 mmol) and CuSO_4_ (0.01 equiv., 20 mg, pentahydrate) was added to the mixture. Imidazole-1-sulfonyl azide H_2_SO_4_-salt [34] (1.2 equiv., 1.3 g, 4.9 mmol) was added under vigorous stirring. The blue-colored reaction mixture was stirred vigorously at room temperature (RT) for 16 h. After overnight stirring, the obtained green-colored reaction mixture was concentrated in vacuum. The residue was dissolved in water (80 mL) and acidified with concentrated HCl. The product was extracted with EtOAc (2 × 60 mL). The collected organic layer was dried (MgSO_4_), filtered, and concentrated to give azido acid as yellow-brown oil. Yield: 1.36 g. Analysis performed by ^1^H- and ^13^C-NMR, IR, LC-MS confirmed the identity of the product. Thin layer chromatography (TLC) (R_f_ = 0.45, DCM/MeOH/HOAc 92:8:0.1 *v*/*v*/*v*) confirmed purity.

#### 2.1.2. Bis(tert-butyl)-l-glutamyl 4-Nitrophenyl Carbamate

Activated 4-nitrophenyl carbamate was prepared by treatment with 4-nitrophenyl chloroformate in the presence of DIPEA in DCM, as described previously [35,36]. In short, chloroformate (686 mg, 3.3 mmol) was dissolved in DCM (100 mL, 4 Å), placed under N_2_ atmosphere, and cooled to 0 °C in an ice–water bath. Bis(*tert*-butyl)-l-glutamyl hydrochloride (888 mg, 3 mmol) was dissolved in DCM, followed by DIPEA (628 µL, 3.6 mmol). This solution was added dropwise to the chloroformate and stirred vigorously for 1 h. The solvent was evaporated in vacuum and the residue was re-dissolved in EtOAc. Organic layer was washed with 1 M KHSO_4_ and dried over MgSO_4_, filtered, and concentrated. The product was obtained as clear pale-yellow oil in 75% yield (0.956 g).

#### 2.1.3. Solid Phase Peptide Synthesis (SPPS)

Peptides were prepared via manual SPPS according to Fmoc/*t*Bu-chemistry protocols [37]. DMF, DCM, piperidine/DMF (1:4 *v*/*v*), and DIPEA were dried over molecular sieves (4 Å). Fmoc-deprotection was performed using piperidine/DMF (1:4 *v*/*v*) for 3 × 15 min, followed by repeated washing with DMF. Each amino acid was coupled sequentially in a four-fold excess, compared to resin loading, using HBTU (4 equiv.) and DIPEA (8 equiv.) in DMF for 120 min at room temperature. Prior to cleavage from the resin, the peptide resin was washed with DCM and dried in vacuum. Cleavage from the resin was performed by treatment with TFA/TIPS/H_2_O (95:2.5:2.5 *v*/*v*/*v*) for 2 h at room temperature. The resin was filtered off and washed with TFA. The filtrate was added dropwise to cold diethyl ether in a centrifuge tube, thoroughly agitated, and centrifuged. After decantation, the precipitate was washed with diethyl ether and dried. The crude peptide was dissolved in aqueous acetonitrile, filtered, and purified by preparative reversed-phase high-performance liquid chromatography (RP-HPLC). Fractions containing the product were pooled and lyophilized. All peptides showed purity above 95% according to HPLC-UV at 254 nM.

#### 2.1.4. Azido-l-Tyr-(9-amino-4,7-dioxanonanoyl)-d-Phe-l-Gln-l-Trp-l-Ala-l-Val-l-Gly-l-His-(3S,4S)Sta-l-Leu-NH_2_) (**3**)

Peptide azide **3** was synthesized from precursor peptide **2** by SPPS on Rink MBHA amide (Knorr resin, loading: 0.7 mmol/g) (Iris Biotech GmbH, Germany), using 2-azido-l-Tyr(tBu)-OH (**1**) as the N-terminal building block [38]. Yield: 30 mg (21 µmol). Calculated mass: 1460.76 (C_71_H_100_N_18_O_16_); observed: 1461.764 [M + H]^+^, 1483.746 [M + Na]^+^, 1499.720 [M + K]^+^.

#### 2.1.5. PSMA-617 alkyne (**4**)

Propynoyl-tranexamyl-l-2-Nal-ε-l-Lys-ureido-l-Glu (**4**) was synthesized as described previously [30,39], with bis(*tert*-butyl)-l-glutamyl 4-nitrophenyl carbamate as the isocyanate precursor, and propynoic acid as the N-terminal amino acid. Scale: 40 µmol. Expected [M − H]^−^: 706.309; observed: 706.308 [M − H]^−^.

#### 2.1.6. PSMA-617 alkyne (**5**)

Similarly, dec-9-ynoyl-tranexamyl-l-2-Nal-ε-l-Lys-ureido-l-Glu (**5**) was synthesized via SPPS using dec-9-ynoic acid as the N-terminal amino acid. Scale: 120 µmol. Formula: C_43_H_59_N_5_O_10_. Expected [M − H]^−^: 804.4184; observed: 804.419 [M − H]^−^.

#### 2.1.7. PSMA-617 alkyne (**6**)

4,7,-dioxa-dec-9-ynoyl-tranexamyl-l-2-Nal-ε-l-Lys-ureido-l-Glu (**6**) was synthesized via SPPS using 4,7,-dioxa-dec-9-ynoic acid as the N-terminal linker. Scale: 113 µmol. Expected [M − H]^−^: 808.3769; observed: 808.379 [M − H]^−^.

#### 2.1.8. BO530

Heterodimer BO530 was synthesized from azide **3** (10 mg, 6.8 µmol) and alkyne **6** (5.54 mg, 6.8 µmol) according to Cu(I)-catalyzed alkyne/azide cycloaddition [40,41]. A glass vial (4 mL) was charged with peptide precursors, followed by 0.5 mL DMF and 0.25 mL H_2_O. Cu(II)SO_4_-sol. was added to Na-ascorbate sol. and mixed. When the pre-activated Cu-sol. turned yellow, it was added to the peptide solution. The reaction vial was capped, and the reaction mixture vigorously stirred for 16 h at RT. The reaction was monitored by LC-MS. After dilution in MeCN/H_2_O to about 1 mL, the crude product was filtered and purified using preparative C18. Isolated product fractions were analyzed, pooled, and freeze-dried. Yield: 5.4 mg (2.4 µmol). Expected [M + H]^+^: 2271.14902; observed: 2271.148 [M + H]^+^, 2293.130 [M + Na]^+^, 2309.104 [M + K]^+^.

#### 2.1.9. BO535

Similarly, heterodimer BO535 was synthesized from azide **3** (9.4 mg, 6.3 µmol) and alkyne **5** (5.1 mg, 6.3 µmol), in an isolated yield of 3.4 mg (1.5 µmol). Expected [M + H]^+^: 2267.19049; observed: 2267.190 [M + H]^+^, 2289.172 [M + Na]^+^, 2305.146 [M + K]^+^.

#### 2.1.10. BO536

Following a similar procedure, heterodimer BO536 was synthesized from azide **3** (9.9 mg, 6.8 µmol) and alkyne **5** (4.7 mg, 6.8 µmol), in an isolated yield of 4.3 mg (2.0 µmol). Expected [M + H]^+^: 2169.08094, observed: 2169.080 [M + H]^+^, 2191.062 [M + Na]^+^, 2207.036 [M + K]^+^.

### 2.2. Radiolabeling and Products’ Characterization

The heterodimers were radiolabeled with I-125 using Pierce Iodination Reagent (IodoGen) (Thermo Fisher Scientific, Waltham, MA, USA). LoBind Eppendorf tubes were coated with IodoGen (50 µg) as previously described [42]. PBS (40 µL) was added to the reaction tube, followed by I-125 and the peptide as previously described [42]. The reaction was allowed to proceed for 15 min at room temperature with occasional slight agitation and terminated by transferring the reaction mixture to a new tube. The reaction mixture was analyzed by reversed-phase HPLC (Hitachi Chromaster, Luna C18 column (5 µm, 100 Å, 150 × 4.6 mm, Phenomenex, Værløse, Denmark)) using a gradient from 30% to 50% acetonitrile (0.1% *v*/*v* TFA) in water over 10 min. Purification was performed under similar conditions with a gradient from 30% to 50% acetonitrile over 20 min. The product fraction was collected and solvent removed using a Sep-Pak C8 Vac 1cc cartridge (Waters Corporation, Milford, MA, USA). The product was eluted in 90% *v*/*v* ethanol in water (120 µL). 

The stability of the radiolabeled peptides was tested in human serum at 37 °C up to 24 h of incubation. At predetermined time points, fractions of 20 µL were mixed with acetonitrile (20 µL), the mixture was centrifuged, and the supernatant was analyzed by instant thin layer chromatography (ITLC) eluted with 0.2 M citric acid.

The octanol–water distribution coefficient was determined as described earlier [43]. Briefly, 500 µL of Milli-Q water and 500 µL of *n*-octanol were added in an Eppendorf tube containing radiolabeled peptide. The tube was vortexed for 2 min and centrifuged for 20 s. Fractions of each phase were collected and their activity was measured.

### 2.3. In Vitro Characterization

An in vitro binding specificity assay was performed using PC-3 (specificity to GRPR), LNCaP (specificity to PSMA) and PC-3pip (specificity to GRPR and PSMA) cells. Cells in six-well plates (5 × 10^5^ cells/well) were pre-treated with 200 nM of either NOTA-PEG_4_-RM26 (to block GRPR) or PSMA-617 (ABX, Radeberg, Germany) (to block PSMA) for 10 min at room temperature. The radiolabeled heterodimer (1 nM) was added to all wells (including non-treated sets) and the plates were incubated for 1 h at 37 °C. After media aspiration, the wells were treated with trypsin–EDTA (Biochrom AG, Berlin, Germany). The detached cells were measured for the activity content.

Half maximal inhibitory concentration (IC_50_) values were determined according to Varasteh et al. [25]: for GRPR in the concentration range of 0–270 nM of the heterodimer using PC-3 cells and 1 nM of [^111^In]In-NOTA-PEG_4_-RM26, and for PSMA in concentration range of 0–1620 nM of the heterodimer using PC-3pip cells and 1 nM of [^111^In]In-PSMA-617 (GRPRs were blocked with 200 nM of unlabeled NOTA-PEG_4_-RM26). Cells were incubated at 4 °C for 5 h. After incubation, the cells were detached with trypsin–EDTA and the activity in cell samples was measured. Data were plotted using nonlinear regression.

The cellular retention of activity after interrupted incubation was carried out on PC-3, LNCaP, and PC-3pip cells. A concentration of 20 nM of each radiolabeled heterodimer was added to the cells and the dishes were incubated at 4 °C for 2 h. The media was aspired, and the cells were further incubated with complete media at 37 °C. At predetermined time points, the media was collected, and the cells were detached with trypsin–EDTA and collected. The radioactivity content of each collected fraction was measured.

### 2.4. In Vivo Characterization

All in vivo experiments were carried out on BALB/c nu/nu mice provided by Scanbur A/S (Sweden). PC-3, LNCaP, and PC-3pip tumor xenografts were established by implanting 5 × 10^6^ cells/mouse for PC-3 and PC-3pip xenografts, and 10 × 10^6^ cells for LNCaP xenografts. The in vivo experiments were carried out when palpable tumors were observed. Animal weight at the moment of experiment was 20 ± 2 g, 21 ± 3 g, 14.5 ± 0.8 g, and average tumor size was 0.2 ± 0.1 g, 0.05 ± 0.04 g, 0.4 ± 0.1 g for PC-3, LNCaP, and PC-3pip models, respectively.

In vivo specificity for the radiolabeled heterodimers was tested on mice bearing PC-3 or LNCaP xenografts. The mice were injected with 40 pmol (30 KBq) of the radiolabeled heterodimer in 1% bovine serum albumin (BSA) in PBS. For the mice in the blocked group, 7.5 nmol of either unlabeled NOTA-PEG_4_-RM26 or PSMA-617 was added to the radiolabeled heterodimer in order to block GRPR in PC-3 xenografted mice or PSMA in LNCaP xenografted mice, respectively. Animals were sacrificed by heart punctures exsanguination after pre-injection of Ketalar–Rompun solution (10 mg/mL Ketalar and 1 mg/mL Rompun; 20 μL solution/gram of body weight). The mice were dissected 1 h post-injection (pi) and the organs of interest were collected. The activity uptake in organs was measured.

Biodistribution studies for [^125^I]I-BO530 were conducted on mice bearing PC-3pip tumor xenografts. The mice were injected with 40 pmol (30 KBq) of [^125^I]I-BO530 in 1% BSA/PBS. The radioactivity uptake in organs was studied 1, 3, 24, and 72 h pi as described above.

Mice bearing PC-3pip xenografts were injected with 40 pmol (500 KBq) of [^125^I]I-BO530 in 1% BSA/PBS. Whole body SPECT/CT scans were performed using nanoScan^®^ SPECT•CT (Mediso Medical Imaging Systems, Budapest, Hungary) at 3, 24, and 72 h pi. CT scans were acquired at the following parameters: 50 keV, 670 μA, 480 projections, 5 min acquisition time; SPECT scans were carried out using I-125 energy window (25.56 keV–31.24 keV), 256 × 256 matrix, 1 h acquisition time. The CT raw data were reconstructed using Nucline 2.03 Software (Mediso Medical Imaging Systems, Budapest, Hungary). SPECT raw data were reconstructed using Tera-Tomo™ 3D SPECT (Mediso Medical Imaging Systems, Budapest, Hungary).

## 3. Results

### 3.1. Heterodimers Production, Radio-Iodination, and Characterization

**Figure 2:**
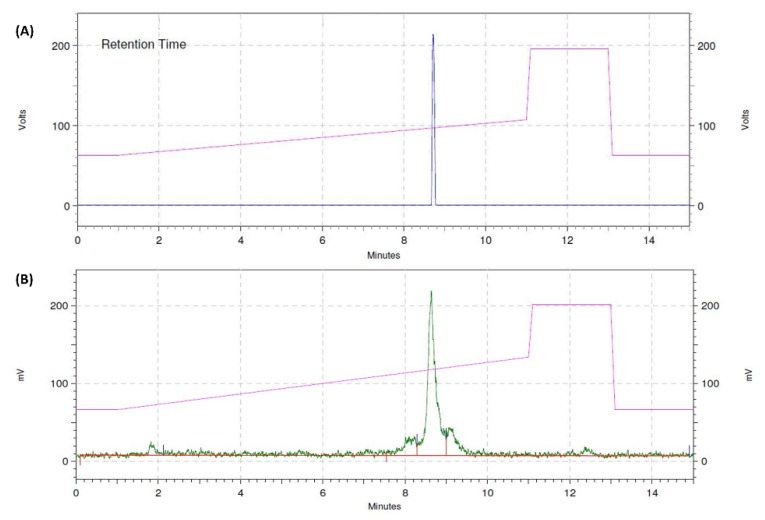
The representative UV chromatogram of the non-labeled BO530 (**A**) and the radio chromatogram of BO530 after radiolabeling with I-125 (**B**).

The heterodimers were successfully synthesized and radiolabeled with I-125 with radiochemical yields 69% ± 1%, 73% ± 4%, 73% ± 3% for BO530 (Figure 2), BO535, and BO536, respectively (determined by HPLC, molar activities of 0.9 MBq/nmol). The yields decreased to 45% ± 1% for BO530 when the molar activities were increased up to 20 MBq/nmol. High purity (at least 95%, determined by HPLC) was achieved after HPLC purification for all radiolabeled heterodimers. The radiolabeled heterodimers were stable in human serum when incubated at 37 °C up to 24 h, minimal release of free iodine (<5%, within method accuracy) was detected when samples were analyzed by ITLC. The octanol–water distribution coefficient showed logD values of −0.52, 1.07, and 0.22 for [^125^I]I-BO530, [^125^I]I-BO535, and [^125^I]I-BO536, respectively.

### 3.2. In Vitro Characterization of Radio-Iodinated Heterodimers

**Figure 3:**
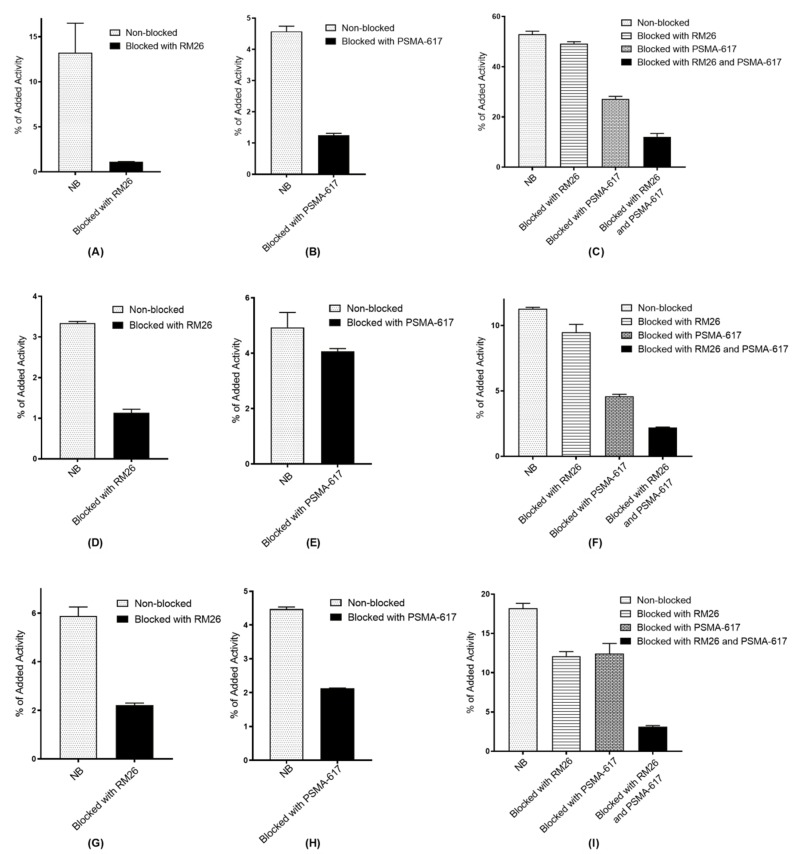
In vitro specificity test for [^125^I]I-BO530 (**A**–**C**), [^125^I]I-BO535 (**D**–**F**), and [^125^I]I-BO536 (**G**–**I**). GRPR in the blocked group were pre-saturated by addition of 200-fold molar excess of non-labeled RM26, PSMA in the blocked group were pre-saturated by addition of 200-fold molar excess of non-labeled PSMA-617. Data are presented as average ± standard deviation.

The in vitro binding specificity test (Figure 3) demonstrated a significant difference in activity uptake between the non-blocked cells compared to the cells which receptors were blocked with excess of non-labeled ligand prior to the addition of the radiolabeled heterodimer. The results of the test demonstrated that all radiolabeled heterodimers bound specifically to GRPR in PC-3 and PSMA in LNCaP cells. When tested in PC-3pip cells (GRPR and PSMA positive), activity uptake in cell samples blocked with both non-labeled ligands was significantly lower than the uptake in the samples blocked with one ligand.

**Figure 4:**
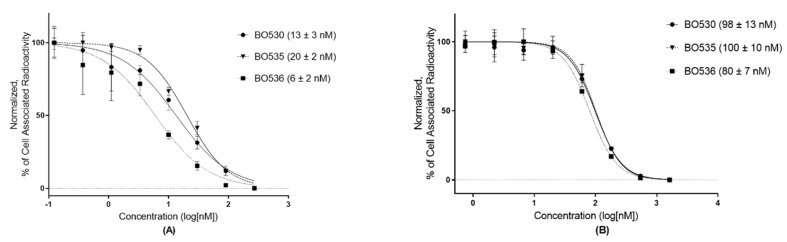
Binding inhibition of [^111^In]In-NOTA-PEG_4_-RM26 (**A**) and [^111^In]In-PSMA-617 (**B**) with BO530, BO535, and BO536 tested using PC-3 (**A**) and PC-3pip, GRPRs were blocked with 200 nM of unlabeled RM26 (**B**). Data are presented as mean value ± standard deviation.

The half-maximal inhibitory concentrations (IC_50_) were determined for the non-iodinated heterodimers by using either [^111^In]In-NOTA-PEG_4_-RM26 as the binding competitor to GRPR, or [^111^In]In-PSMA-617 as the binding competitor to PSMA. The values (Figure 4) of the heterodimers to GRPR were in the low nanomolar range, while to PSMA the values were around 100 nM. 

**Figure 5:**
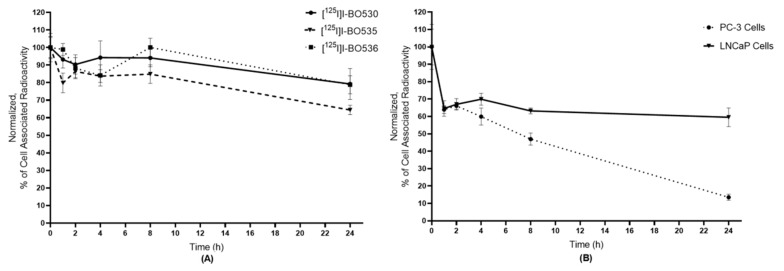
Cellular retention of activity (normalized to 0 h) after interrupted incubation for [^125^I]I-BO530, [^125^I]I-BO535, and [^125^I]I-BO536 on PC-3pip cells (**A**), and for [^125^I]I-BO530 on PC-3 and LNCaP (**B**) cells. Data are presented as mean value ± standard deviation.

The cellular retention of activity after interrupted incubation on PC-3pip cells (Figure 5A) showed minimal release of the cell associated activity after 8 h of incubation. At the 24 h, cell-associated activity decreased by 20% for [^125^I]I-BO530 and [^125^I]I-BO536, and 35% for [^125^I]I-BO535. To test the role of antigen/ligand interaction in retention, retention of [^125^I]I-BO530 was additionally studied on PC-3 and LNCaP cells (Figure 5B). The cellular retention of [^125^I]I-BO530 on PC-3 cells (GRPR positive) showed constant release of cell-associated activity (14% ± 2% at 24 h of incubation). On LNCaP cells (PSMA positive) cell-associated activity rapidly decreased during the first hour and remained stable between 1 and 24 h. Cell associated activity was 60% from initial at 24 h. Additionally, we applied a common assay allowing discrimination of membrane-bound and internalized activity for radio-iodinated heterodimers [27] and found that it was not able to quantitatively detach heterodimers from the cell surface after incubation of cells with heterodimers on ice (data not shown).

### 3.3. In Vivo Characterization of Radio-Iodinated Heterodimers

In vivo targeting specificity was tested for all the radiolabeled heterodimers in mice bearing either PC-3 (GRPR positive) or LNCaP (PSMA positive) xenografts. In vivo binding of radio-iodinated heterodimers to GRPR and PSMA was blocked by co-injection of non-labeled 7.5 nmol of either unlabeled RM26 or PSMA-617, respectively. It was found that in mice bearing PC-3 xenografts (Table 1) [^125^I]I-BO530 showed significant difference in activity uptake by tumors and GRPR-expressing organs (pancreas and stomach) between the non-blocked and blocked groups. Heterodimers [^125^I]I-BO535 and [^125^I]I-BO536 failed to demonstrate significant difference in activity uptake in GRPR positive tumors between the blocked and non-blocked groups. However, activity uptake in GRPR expressing normal organs (e.g.*,* pancreas) was significantly lower in groups co-injected with non-labeled RM26 for both heterodimers. In mice bearing LNCaP xenografts (Table 2), only [^125^I]I-BO530 and [^125^I]I-BO536 demonstrated significant difference in tumor activity uptake between the non-blocked and blocked groups while the significant difference in kidney activity uptake (PSMA expressing) was shown for all tested radiolabeled heterodimers. Activity uptake in lungs after injection of radio-iodinated heterodimers depended on the xenograft model, e.g., for [^125^I]I-BO530 activity uptake in PC-3 xenografted mice was significantly higher than uptake in LNCaP xenografted mice. Co-injection of non-labeled RM26 or PSMA-617 influenced activity uptake in a mixed pattern, while activity uptake of [^125^I]I-BO530 increased in both models, uptake of [^125^I]I-BO535 was blocked only by co-injection of PSMA-617, and was not changed for [^125^I]I-BO536.

**Figure 6:**
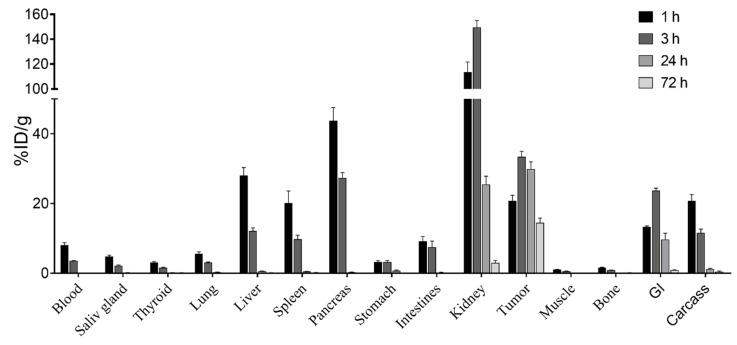
Biodistribution of [^125^I]I-BO530. Balb/c nu/nu mice with PC-3pip (GRPR and PSMA positive) xenografts were injected with 40 pmol (30 KBq) of labeled heterodimer. Data are presented as mean value ± standard deviation.

A biodistribution study of [^125^I]I-BO530 over time (Figure 6) was performed in mice bearing PC-3pip xenografts. Elevated activity uptake was found 1 h pi in several organs including blood, kidneys, and tumors. Activity uptake in all healthy organs decreased with time, except in the kidneys, and 24 h pi activity uptake in normal organs decreased below 1% ID/g (except kidneys). The activity uptake in kidneys decreased five-fold at this time point. The activity uptake in tumors increased significantly between 1 and 3 h pi without noticeable washout up to 24 h pi. At the latest tested time point (72 h pi) the activity uptake in tumors decreased two-fold, but was much higher than in normal organs.

**Figure 7:**
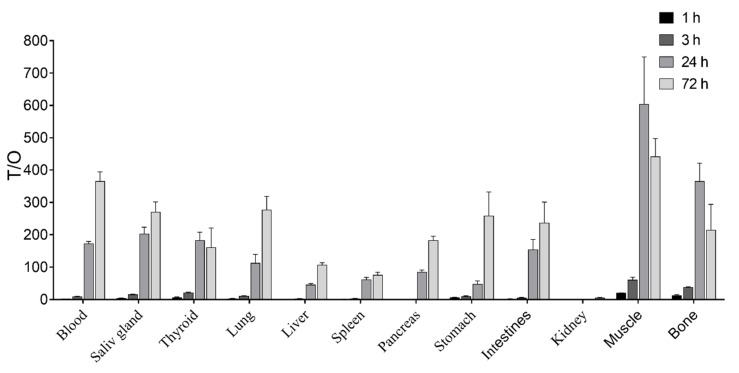
Tumor-to organ ratios for [^125^I]I-BO530. Balb/c nu/nu mice with PC-3pip (GRPR and PSMA positive) xenografts were injected with 40 pmol (30 KBq) of labeled heterodimer. Data are presented as mean value ± standard deviation.

As the uptake in organs decreased over time and the tumor uptake remained high, the tumor-to-organ ratios (T/O) increased with time (Figure 7). Tumor-to-kidney ratios were 1.2 ± 0.3 and 5 ± 2 and tumor-to-liver 46 ± 7 and 108 ± 13, 24 h and 72 h pi, respectively. Activity concentration in tumors was at least 100-fold higher than in normal organs relevant in context of prostate cancer (blood, intestines, muscle, bones) 24 h pi. The tumor-to-normal-organ ratios further increased with time, except for muscle and bones.

**Figure 8:**
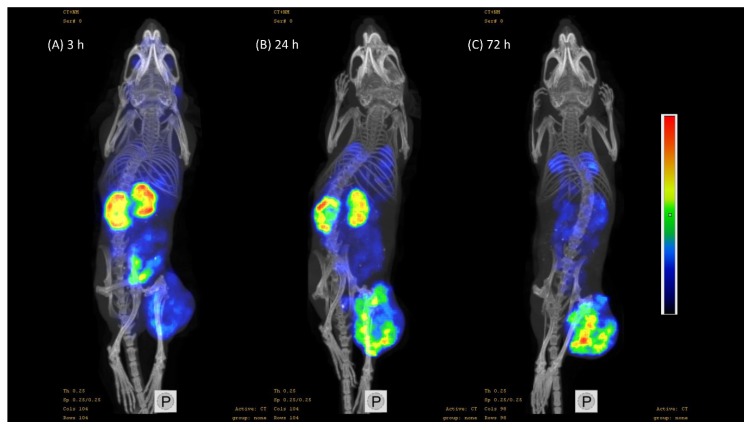
MicroSPECT-CT imaging of [^125^I]I-BO530 at 3 (**A**), 24 (**B**), and 72 h pi (**C**). Balb/c nu/nu mice with PC-3pip (GRPR and PSMA positive) xenografts were injected with 40 pmol (500 KBq) of labeled heterodimer.

The SPECT/CT images of mice bearing PC-3pip tumors taken 3, 24, and 72 h pi of [^125^I]I-BO530 (Figure 8) corroborated with the data of ex vivo measurements. High contrast images were acquired. Activity uptake in tumoru and kidneys was visualized, however, 72 h pi tumor activity uptake dominated the image.

## 4. Discussion

Considerable efforts have been made in the past decades to target the prostate cancer cell markers GRPR and PSMA. An approach to target both prostate cancer cell markers with a single tracer is highly attractive. Such a tracer could cover the whole spectrum of the disease by targeting GRPR which is highly overexpressed in the early stages and PSMA that is predominantly overexpressed in the late, androgen independent, stages of the disease, thus potentially increasing the sensitivity towards detecting prostate cancer and its distant metastases. The first GRPR/PSMA-targeting heterodimers were reported few years ago [22,23]. Later one more GRPR/PSMA bispecific heterodimer was reported [44,45]. All these heterodimers were based on agonist towards GRPR and radiolabeled with radiometals with the aim of improving activity retention in tumors. We have since designed GRPR/PSMA bispecific heterodimers suitable for radio-iodination with the aim to apply them in theranostics purposes. To prevent rapid leakage of activity from tumor cells after internalization we chose a GRPR antagonist. The availability of radio-iodinated GRPR/PSMA bispecific heterodimers could provide an advantage for theranostics applications as they could be used for imaging of prostate cancers by SPECT and PET cameras with I-123 and I-124 labels, respectively, and for therapeutic purposes labeled with I-131. Theranostics applications of radiotracers can be achieved when the tracers have a long retention time in the targeted cells and low uptake and/or rapid clearance from healthy tissue in order to maximize the delivered dose and to provide high contrast between the tumor and background in the images. 

The previous efforts done to target both GRPR and PSMA with a single tracer have used GRPR targeting motifs possessing agonistic functions. Owing to the internalization characteristics of GRPR agonists and triggered by the potential side effects and extensive downregulation of GRPR, we have incorporated antagonistic RM26 as the GRPR targeting motif in our heterodimers in order to maximize tumor uptake and slow down internalization via this receptor. The PSMA inhibitor PSMA-617 is known to possess favorable kinetics and was therefore used as the PSMA targeting motif in the heterodimers. The tyrosyl was incorporated as the binding site for radio-iodine. The synthesized variants of heterodimer differed in the linkers used to couple PSMA binding moiety and hence the lipophilicity. The less lipophilic variant as expected contained hydrophilic PEG linker (BO530), while the variant containing (CH_2_)_8_-linker was the most lipophilic (BO535), as expected. Good radiochemical yields were achieved when the heterodimers were radiolabeled with I-125 using a mild oxidant (IodoGen). All the radiolabeled heterodimers bound specifically in vitro to both GRPR and PSMA on PC-3 (GRPR positive), LNCaP (PSMA positive), and PC-3pip (GRPR and PSMA positive) cells. All three heterodimers had higher affinity for GRPR than for PSMA. The IC_50_ values for the three heterodimers towards GRPR were in the low nanomolar range while the IC_50_ values for PSMA were around 100 nM; [^125^I]I-BO536 appeared to be the most potent of the three heterodimers with the best IC_50_ values for both GRPR and PSMA. 

It has previously been demonstrated that radio-iodinated glu–urea derivatives targeting PSMA could have long retention over time [46]. The radio-iodinated heterodimers in this study demonstrated unprecedently high (for radio-iodinated short peptide) cellular retention when tested in cells expressing both molecular targets (PC-3pip). Typically, activity retention of radiohalogenated peptides and short proteins is pure due to non-residualizing properties of radiocatabolites [47,48]. To further understand the observed results, we investigated the cellular retention of [^125^I]I-BO530 in PC-3 (GRPR positive) and LNCaP (PSMA positive) cells. The results showed that [^125^I]I-BO530 cleared more rapidly in PC-3 cells while it was highly retained by LNCaP cells over time. This indirectly indicated that cellular processing of the new heterodimers is mainly determined by interaction with PSMA. For comparison, GRPR agonists [^18^F]F-FB-[Lys^3^]BBN and [^18^F]F-FB-Aca-BBN(7–14) were rapidly internalized (maximum for internalized activity was found at 15 min) followed by excretion of 75%–85% of internalized activity within 3 h [45].

In vivo specificity of the three radiolabeled heterodimers was studied in mice bearing PC-3 or LNCaP xenografts. Only [^125^I]I-BO530 demonstrated in vivo specificity to both targets. While [^125^I]I-BO536 had the best affinities for both targets, it did not demonstrate in vivo specificity for GRPR in tumors. Also [^125^I]I-BO535 failed to demonstrate in vivo specificity to both targeted xenografts despite its prominent affinity for GRPR and PSMA. However, their uptake in the pancreas and kidneys showed significant differences between the non-blocked and blocked groups. Partially this finding could be explained by high lipophilicity of [^125^I]I-BO535 that manifested in high activity uptake in blood and liver and low uptake in kidneys in comparison with the less lipophilic heterodimer. Off-target sequestering of radiotracer could have resulted in lower bioavailability and therefore lower uptake in tumors which additionally have worse penetration. The activity uptake in the GRPR tumors was significantly higher for [^125^I]I-BO530 (4.3% ± 0.6%) than for [^125^I]I-BO535 (2.2% ± 0.6%) and [^125^I]I-BO536 (3.0% ± 0.3%) and was in agreement with data reported for heterodimers labeled with Ga-68 and Lu-177 reported previously [24,45]. The activity uptake for [^125^I]I-BO530 (10% ± 3%) and [^125^I]I-BO536 (11% ± 3%) in LNCaP tumors was significantly higher than for [^125^I]I-BO535 (3% ± 2%) and also in agreement with previously reported heterodimers labeled with radiometals. 

The aforementioned results for [^125^I]I-BO530 led to its selection for further evaluation in PC-3pip xenografts expressing both GRPR and PSMA. The biodistribution results were in good agreement with the in vitro observations. The biodistribution study showed a high tumor uptake at 1 h pi which continued to increase up to 3 h pi. Interestingly the tumor uptake remained stable up to 24 h pi. At 72 h pi, the tumor uptake decreased only 2-fold demonstrating promising indications for the potential use of [^125^I]I-BO530 for therapy. The initial blood uptake was high which reflected in a later increase in the tumor uptake indicating that the high blood uptake is not owed to radiocatabolites. The elevated kidney uptake is owed to the normal expression of PSMA in kidneys; however the renal uptake decreased more rapidly than the uptake in tumors. For comparison, ratio of activity uptake in kidneys and in tumors for [^125^I]I-BO530 was better than reported for ^177^Lu-PSMA-617 that nowadays is actively used for treatment of prostate cancer patients: initial uptake of ^177^Lu-PSMA-617 in kidneys was at least 10-fold higher than in tumors [30].The activity uptake in the intestines, muscle, and bone was low and almost diminished at 24 h pi. The area under the curve (AUC, the rough estimation of the dose delivered to the organ) was 10-fold higher for tumor than for blood but more than two-fold higher for the kidneys than for the tumors, indicating that the dose delivered to the kidneys could be unacceptably high and therefore more work needs to be done to decrease the elevated kidney uptake. There are several approaches that allow to decrease renal activity uptake of radiolabeled peptides that could be investigated [49].

The tumor-to-organ ratios substantially increased with time, reaching satisfactory ratios for the majority of organs 24 h pi. At 72 h pi, the ratios further increased for most organs as the activity continued to clear from normal organs. It has been established that the most common site of prostate cancer metastases is the lymph nodes and bones, followed by liver, and the lungs [50]. The tumor-to-organ ratios achieved after injection of [^125^I]I-BO530 at 24 and 72 h pi were high with relation to the main sites of prostate cancer metastases. The mice were imaged at 3, 24, and 72 h pi and the images were in good agreement with the ex vivo findings providing a high contrast between the tumor and background 24 and 72 h pi.

## 5. Conclusion

In conclusion, in this study we reported the development of radio-iodinated GRPR/PSMA-targeting bispecific heterodimer [^125^I]I-BO530 for theranostic applications in prostate cancer. The synthesized compound BO530 was radio-iodinated with high radiochemical yields and high radiochemical purities at high molar activities. The heterodimer showed in vivo targeting specificity to GRPR and PSMA and long activity retention in tumors while being cleared from normal organs providing images with high contrast. The findings demonstrate that while [^125^I]I-BO530 is a promising agent for the theranostics of prostate cancer, continued development towards improved radiotracers with reduced kidney uptake is necessary.

## Figures and Tables

**Table 1:** In vivo specificity GRPR tested 1 h post injection (pi) of [^125^I]I-BO530, [^125^I]I-BO535, and [^125^I]I-BO536 (%ID/g)**.** Balb/c nu/nu mice with PC-3 xenografts were injected with 40 pmol (30 KBq) of labeled heterodimer (non-blocked group). For the specificity test, the heterodimer was co-injected with 7.5 nmol of unlabeled RM26 (blocked group). Data are presented as mean value ± standard deviation.

Heterodimer	[^125^I]I-BO530		[^125^I]I-BO535		[^125^I]I-BO536	
Organ	Non-blocked	Blocked	Non-blocked	Blocked	Non-blocked	Blocked
Blood	5 ± 1	4.5 ± 0.6	8 ± 1	8.4 ± 0.7	5.0 ± 0.7	6 ± 2
Tumor	4.3 ± 0.6	2.3 ± 0.3 ^1^	2.2 ± 0.6	2.2 ± 0.2	3.0 ± 0.3	2.6 ± 0.2
Saliv. Gl.	2.0 ± 0.3	1.7 ± 0.2	2.2 ± 0.5	2.4 ± 0.3	2.7 ± 0.3	2.8 ± 0.5
Thyroid	5 ± 1	3.1 ± 0.3 ^1^	4.4 ± 0.9	4.3 ± 0.7	3.7 ± 0.6	3.8 ± 0.8
Lung	20 ± 7	35 ± 4 ^1^	10 ± 2	10 ± 2	13 ± 4	15 ± 7
Liver	23 ± 3	23 ± 4	27 ± 4	32 ± 1	23 ± 3	26 ± 5
Spleen	8 ± 2	6.4 ± 0.4	3.2 ± 0.7	3.6 ± 0.4	12 ± 1	11.6 ± 0.8
Pancreas	19 ± 4	5 ± 1 ^1^	4 ± 1	1.7 ± 0.1 ^1^	8 ± 1	2.7 ± 0.4 ^1^
Stomach	2.2 ± 0.2	1.4 ± 0.2 ^1^	1.5 ± 0.3	1.6 ± 0.3	2.2 ± 0.5	1.8 ± 0.3
Intestines	4 ± 1	2.8 ± 0.7	3.2 ± 0.8	3.3 ± 0.6	3.8 ± 0.5	4 ± 1
Kidneys	66 ± 18	61 ± 16	12 ± 3	14 ± 2	51 ± 6	48 ± 9
Muscle	0.7 ± 0.2	0.6 ± 0.1	0.7 ± 0.2	1.0 ± 0.3	0.8 ± 0.1	0.8 ± 0.1
Bone	0.8 ± 0.2	0.8 ± 0.1	0.9 ± 0.2	1.2 ± 0.3	0.8 ± 0.2	0.9 ± 0.3

^1^ Differences were significant (*p* < 0.05) between non-blocked and blocked groups.

**Table 2:** In vivo specificity to PSMA tested 1 h pi of [^125^I]I-BO530, [^125^I]I-BO535, and [^125^I]I-BO536 (%ID/g). Balb/c nu/nu mice with LNCaP xenografts were injected with 40 pmol (30 KBq) of labeled heterodimer (non-blocked group). For the specificity test, the heterodimer was co-injected with 7.5 nmol of unlabeled PSMA-617 (blocked group). Data are presented as mean value ± standard deviation.

Heterodimer	[^125^I]I-BO530		[^125^I]I-BO535		[^125^I]I-BO536	
Organ	Non-blocked	Blocked	Non-blocked	Blocked	Non-blocked	Blocked
Blood	4 ± 1	4 ± 1	6 ± 1	6 ± 1	8 ± 3	6.0 ± 0.9
Tumor	10 ± 3	2 ± 2 ^1^	3 ± 2	2 ± 2	11 ± 3	1.4 ± 0.7 ^1^
Saliv. Gl.	1.7 ± 0.7	1.5 ± 0.3	1.7 ± 0.4	1.5 ± 0.4	3.1 ± 0.8	1.9 ± 0.2 ^1^
Thyroid	2.6 ± 0.5	3.5 ± 0.9	3.3 ± 0.5	3.4 ± 0.6	4 ± 1	3.4 ± 0.6
Lung	5.6 ± 0.9 ^1^	46 ± 19	21.5 ± 0.8	14 ± 3 ^1^	5 ± 1	8 ± 1 ^1^
Liver	29 ± 3	27 ± 1	40 ± 5	37 ± 7	30 ± 9	29 ± 3
Spleen	9 ± 5	6 ± 2	4.5 ± 0.8	3.4 ± 0.8	18 ± 6	3.5 ± 0.8 ^1^
Pancreas	28 ± 6	22 ± 3	7 ± 2	7 ± 2	10 ± 2	7.0 ± 0.7 ^1^
Stomach	1.9 ± 0.4	2.3 ± 0.8	1.1 ± 0.2	1.2 ± 0.2	3 ± 2	3 ± 1
Intestines	7 ± 3	4.4 ± 0.4	3.2 ± 0.8	2.7 ± 0.7	5 ± 1	5 ± 2
Kidneys	56 ± 22	13.1 ± 0.7 ^1^	6.8 ± 0.9	3.8 ± 0.5 ^1^	63 ± 20	19 ± 3 ^1^
Muscle	0.5 ± 0.1	0.6 ± 0.1	0.4 ± 0.1	0.4 ± 0.1	0.8 ± 0.2	0.6 ± 0.1
Bone	0.6 ± 0.1	0.8 ± 0.4	0.7 ± 0.1	0.7 ± 0.3	0.8 ± 0.1	0.9 ± 0.3

^1^ Differences were significant (*p* < 0.05) between non-blocked and blocked groups.

## Appendix A. Cell Lines, Chemicals and Equipment

Prostate carcinoma cell lines PC-3 (GRPR positive) and LNCaP (PSMA positive) were purchased from ATCC, USA. Prostate carcinoma PC-3 cells transfected with PSMA PC-3pip (GRPR and PSMA positive) were obtained from Dr. Warren Heston, Cleveland Clinic. PC-3 and PC-3pip cells were maintained in Rosewell Park Memorial Institute (RPMI)-1640 media supplemented with 20% fetal bovine serum (FBS), 1% penicillin-streptomycin (PEST) (100 IU/mL penicillin, 100 µg/mL streptomycin), and 1% 2 mM l-glutamine (l-Glut), all from Biochrom AG, Germany. PC-3pip cells were additionally maintained every other week in RPMI-1640 media supplemented with 20% FBS, 1% PEST, 1% l-Glut, and 20 µg/mL puromycin (Sigma Aldrich, Steinheim, Germany). LNCaP cells were maintained in RPMI-1640 media supplemented with 10% FBS, 1% penicillin-streptomycin, 1% l-glutamine, 1% hydroxyethyl-piperazineethane-sulfonic acid buffer (HEPES) (1 M), and 1% sodium pyruvate (100 mM). All cells were incubated at 37 °C in 5% carbon dioxide gas.

Unless otherwise stated, solvents and reagents were acquired from commercial sources and used without further purification. *N*,*N*-dimethylformamide (DMF) was purchased from Thermo Fischer Scientific, dichloromethane (DCM), trifluoroacetic acid (TFA), acetonitrile (MeCN), and diethyl ether from Sigma Aldrich. The 9-fluorenylmethoxycarbonyl (Fmoc) protected amino acids were purchased from Novabiochem (Merch Millipore, Darmstadt, Germany). The 2-chloro-trityl chloride polystyrene (PS) resin (1.1 mmol/g) and Rink Amide PS resin (0.68 mmol/g) were purchased from IRIS Biotech, Marktredwitz, Germany. Piperidine, *N*,*N*-diisopropylethylamine (DIPEA), *N*-[(1H-benzotriazol-1-yl)(dimethylamino)methylene]-*N*-methylmethanaminium hexafluorophosphate *N*-oxide (HBTU), and triisopropylsilane (TIPS) were purchased from Sigma Aldrich.

Nuclear magnetic resonance (NMR) spectra were recorded on a Varian Mercury Plus at 25 °C; for 1H at 400 MHz and for 13C NMR at 100 MHz. Thin-layer chromatography (TLC) was performed with aluminum sheets coated with silica gel 60-F254 (0.2 mm E. Merck), using UV-light for visualization. Column flash chromatography was performed using silica gel 60 (particle size 0.040–0.063 mm, Sigma-Aldrich). Analytical high performance liquid chromatography (HPLC) was performed on a Dionex UltiMate 3000 HPLC system with a Bruker amaZon SL ion trap mass spectrometer and detection by UV (diode array detector (DAD)) and MS (electrospray ionization (ESI+)), using a Phenomenex Kinetex C18 column (50 × 3.0 mm, 2.6 µm particle size, 100 Å pore size) and a flow rate of 1.5 mL/min. A gradient of H_2_O/CH_3_CN/0.05% HCOOH was used. Water with electrical resistivity of 18.2 MΩ·cm was obtained using a Milli-Q water purification system. Preparative RP-HPLC was performed by UV-triggered (220 or 254 nm) fraction collection with a Gilson HPLC system using a Macherey-Nagel Nucleodur C18 HTec (21 mm × 125 mm, particle size 5 μm), with a H_2_O/MeCN gradient with 0.1% TFA as mobile phase at a flow rate of 10 mL/min. High resolution molecular mass (HRMS) was determined on Waters LCT Premier™ time-of-flight (TOF) mass spectrometer using electrospray ionization (ESI) or Bruker 7 Tesla FT-ICR SolariX mass spectrometer using matrix-assisted laser desorption ionization (MALDI).
